# Does Treatment for Sexual Offending Work?

**DOI:** 10.1007/s11920-021-01259-3

**Published:** 2021-07-01

**Authors:** Nichola Tyler, Theresa A. Gannon, Mark E. Olver

**Affiliations:** 1 School of Psychology, Victoria University of Wellington, PO Box 600, Wellington, 6140 New Zealand; 2 Centre of Research and Education in Forensic Psychology (CORE-FP), School of Psychology, University of Kent, Canterbury, CT2 7NZ UK; 3 Department of Psychology, University of Saskatchewan, Saskatoon, Saskatchewan S7N 5A5 Canada

**Keywords:** Sexual offense treatment, Sexual recidivism, Treatment effectiveness, Sexual offending, Treatment moderators

## Abstract

**Purpose of Review:**

We review and synthesize the literature on the effectiveness of offense-focused treatment for sexual offending. Specifically, we consider whether the extant literature suggests treatment is effective in reducing sexual reoffending and features of effective interventions. We also consider how the design of program evaluations may influence treatment outcomes.

**Recent Findings:**

Recent research suggests that offense-focused psychological treatment for sexual offending shows some level of effectiveness in reducing both sexual and general reoffending. Further, there appear to be key program, individual, and study design features associated with treatment effectiveness.

**Summary:**

Although recent findings paint an optimistic outlook for offense-focused psychological treatment for sexual offending, further high-quality differential studies are needed to fully understand the range of content, delivery, and individual factors associated with successful treatment outcomes so as to establish what works best for whom.

## Introduction

Offense-specific treatment for sexual offending is commonly provided in Western countries with the primary aim of reducing risk of sexual reoffending. Treatment is often psychological and is provided across various settings including the community, forensic mental health hospitals, and prison settings. Nevertheless, although treatment is a common feature of forensic practice for sexual offending, there is much variation across programs in terms of content, structure, and delivery [1]. Given the widespread use of treatment for sexual offending, it is imperative to understand whether such interventions result in meaningful reductions in sexual reoffending, not only for public protection but also to ensure interventions are beneficial for those who volunteer or are mandated to attend them.

The British Ministry of Justice evaluation of the “Core” psychological sexual offender treatment program (SOTP) recently highlighted the importance of understanding “what works” in treating sexual offending [2••]. In this study—which is the largest single study evaluation to date—the reoffending rates for men who completed the “Core” SOTP (*n* = 13,219) in England and Wales (between 2000 and 2012) were compared to those of a propensity score-matched untreated comparison group (*n* = 2562). Over an average 8.2-year follow-up, nonsexual reoffending rates appeared largely similar across the groups. However, sexual reoffending for the treated sample was found to be *higher* than that of the untreated comparison group (10% versus 8%, respectively), representing an absolute increase in sexual reoffending of 2% and a relative increase of 25%. The findings from this study understandably created concern. In short, they suggested that tens of thousands of individuals who had sexually offended and received psychological “treatment” may have been made worse by a program intended to make them better [3]. This study also highlighted the importance of understanding the key ingredients associated with successful treatment for sexual offending.

A previous review of the literature on psychological interventions for sexual offending was reported in this journal in 2013 [4]. Since then, there have been a number of key developments in the field, including the British Ministry of Justice study and one large meta-analysis involving over 40,000 individuals examining the effectiveness of psychological treatment programs for sexual offending [5••]. The purpose of the current article is to provide a recent and updated synthesis of (1) the literature on the effectiveness of offense-specific psychological treatment for reducing sexual reoffending and (2) the factors that may influence the effectiveness of such programs (e.g., program orientation, content, staffing, and individual characteristics). Consideration will also be given to the effect of study design on evaluation outcome, an area that has attracted much attention and debate from researchers in the field both historically [6–9] and more recently [10–13]. Throughout the review, we will specifically focus on the findings from key meta-analyses and important single studies that have contributed to current understanding in the area. Given the vast majority of research and practice has focused on the treatment of adult males who have committed sexual offenses, we will focus on the effectiveness of treatment with this population.

## Effectiveness of Offense-Specific Treatment for Sexual Offending

A number of meta-analyses have been undertaken over the last 20 years that have synthesized outcome evaluations of treatments for sexual offending [5–8, 10, 12, 14–17]. Many of these studies have examined both biological and psychological treatments [8, 10] or sexual offense-specific and more generic psychological treatments [7, 16]. While not all of these meta-analyses have found positive treatment effects [6, 17] , at least seven indicate reductions in sexual reoffending [5, 7, 8, 10, 12, 14, 16].

Lösel and Schmucker’s 2005 meta-analysis included 69 evaluations of biological (e.g., surgical castration and hormonal medication) and psychological treatments for adults and adolescents who had sexually offended (*n* = 22,181) [8]. The treatment groups showed lower rates of sexual (11.1% vs. 17.5%), violent (6.6% vs. 11.8%), and any reoffending (22.4% vs. 32.5%) relative to comparisons. Overall, biological treatments (vs. psychological) were found to produce stronger effects as did treatments designed specifically for sexual offenses. Schmucker and Lösel updated this meta-analysis 10 years later but with an exclusive focus on high-quality studies (i.e., quasi-experimental design with between group equality or above) [12]. Twenty-seven studies (*n* = 10,387) were included in the final meta-analysis, and all of these were psychological interventions. Although low rates of reoffending were noted for the treatment groups, these were smaller than detected in the previous meta-analysis (10.1% vs. 13.7% for sexual reoffending; 32.6% vs. 41.2% for any reoffending).

Most recently, Gannon et al.^1^ conducted the largest meta-analysis to date comprising 41,476 individuals who had sexually offended and including the politically influential British Ministry of Justice “Core” SOTP evaluation [5••]. This meta-analysis examined only offense-specific psychological treatment for sexual offending as well as staff and program variables previously not explored. Although the British Ministry of Justice evaluation was identified as an outlier, when it was included in the analysis, sexual offense programs were found to produce significant reductions across all types of reoffending, most notably in sexual reoffending (9.5% vs. 14.1%) amounting to a relative reduction in sexual reoffending of 32.6%. This meta-analysis also highlighted the key staff and treatment program moderators required for optimal reoffending reductions which we describe in the sections below.

## Factors Influencing Treatment Effectiveness

Given correctional policy makers and practitioners are under increasing pressure to provide interventions that are maximally effective, from both a societal and fiscal perspective, it is crucial to understand the core features or ingredients of treatment that produce the strongest and most sustained effects. Potential moderating factors that have been examined to date can be broadly grouped into (1) program orientation and delivery method (e.g., underlying theory, principles, duration, and modality), (2) program content (e.g., exercises, treatment targets), (3) program staffing, and (4) treatment setting.

### Program Orientation and Delivery Method

Meta-analyses have shown that the underlying theory, principles, dosage, and modality of treatment are associated with differences in treatment outcomes. For example, psychological programs that adhere to the risk (i.e., match treatment intensity to risk level), need (i.e., prioritize dynamic risk factors, aka criminogenic needs, for intervention), and responsivity (i.e., individualize treatments to unique client characteristics) (or RNR) principles of effective rehabilitation [19] show larger reductions for both sexual and general reoffending compared to those that do not adhere to these principles [7]. Psychological interventions that are cognitive behavioral (e.g., CBT) in orientation have also been shown to elicit stronger positive effects [8, 12, 20]. However, it is important to note that the majority of evaluations have focused on CBT-based interventions; therefore, it is difficult to make comparisons with other therapeutic approaches [5, 8, 16].

While strength-based psychological approaches, such as the Good Lives Model [21], are increasingly popular in the criminal justice system, there are almost no longitudinal evaluations examining the effects of these on sexual reoffending [22]. One exception to this is a recent study by Olver, Marshall, Marshall, and Nicholaichuk which presented a retrospective comparative evaluation of two prison-based sexual offense-focused psychological interventions: Correctional Service Canada’s (CSC) SOTP and an early version of the Rockwood treatment program [23•]. Both interventions evaluated were underpinned by a CBT/RNR approach; however, the Rockwood program incorporated strength-based elements, whereas the CSC SOTP program encapsulated more traditional risk reduction/management principles [23–25]. The recipients of both groups were compared to an untreated comparison group on reoffending data obtained from police records which included all charges and convictions over an 8-year average follow-up period. Statistical controls were used to manage key differences across the groups such as baseline risk. While both treatment groups displayed significantly lower rates of sexual and violent reoffending relative to the comparison group, the strength-based Rockwood program generated the lowest sexual reoffending rates (5.4% vs. CSC SOTP 12.6% vs. the comparison group 19.6%). While this is a single study, the results offer some support for the inclusion of strength-based elements in psychological treatment for sexual offending.

Researchers have come to varying conclusions about the impact of treatment length and format on treatment success. For example, Schmucker and Lösel [12] did not find any association between treatment duration (measured by the number of weeks treatment delivered) and reoffending; however, Gannon et al. reported effects according to number of hours delivered [5••]. Reductions in reoffending were observed across all treatment lengths; however, 100- 200 hour programs generated smaller effects than shorter (i.e., less than 100 hours) and longer (i.e., more than 200 hours) programs. Although these two meta-analyses have drawn different conclusions about the influence of treatment length on effectiveness, neither factored in participants’ risk level which may determine the length of treatment received.

A recent review by Day et al. highlighted similar issues with the research on intensity and timing of treatment (i.e., when treatment is delivered during a person’s sentence) [26•]. With respect to intensity, Day et al. concluded that there is much inconsistency in the operationalization of treatment intensity within and across jurisdictions and that there is a need for greater standardization in the calculation of dosage for sexual offense programs. Moreover, with regard to timing of treatment, although Day et al. identified clinical opinion on the optimal timing of treatment, they were unable to identify any studies that had directly examined the effect of treatment timing and therefore concluded that it was not possible to draw firm inferences. Findings for the effect of delivery format are also variable, with Schmucker and Lösel reporting stronger effects for individual and mixed individual/group treatment formats [12] and Gannon et al. finding stronger effects for group-only treatment [5••]. It should be noted, however, that more mixed individual/group treatment format studies were available in Gannon et al.’s meta-analysis (*n* = 18) relative to Schmucker and Lösel’s (*k* = 4). Moreover, while Gannon et al. included only studies of treated adult samples, Schmucker and Lösel also included adolescent treatment outcome studies which featured some individual treatment models (e.g., multisystemic therapy) with large treatment effects. Finally, Thornton (personal communication November 22, 2019) raised the possibility that psychologist presence and/or supervision may moderate the association between modality and outcome; in his examination of the Gannon et al. data, when one of these elements were available, there was little difference in magnitude of effect for group vs. mixed modality; however, when neither elements was available, there was a decidedly larger effect for group modality. Further research is needed to directly compare individual vs. group treatment or mixed individual/group vs. group treatment only on reoffending using direct comparisons (e.g., same intervention delivered in different formats).

### Program Content

Only one meta-analysis to date has directly examined the impact of program content on treatment outcomes. Gannon et al. specifically examined whether the inclusion of behavioral reconditioning procedures for inappropriate sexual arousal impacted treatment effectiveness [5••]. Programs that included some form of behavioral reconditioning (*k* = 23) were associated with superior outcomes (i.e., greater reductions in reoffending) relative to those that did not include this element (*k* = 5) or for whom this element was unknown (*k* = 16). This is a particularly interesting finding given that behavioral reconditioning procedures were not used in the “Core” SOTP evaluated by the Ministry of Justice. Gannon et al. also examined whether or not programs included polygraph testing as part of their protocol. Although only a small number of programs incorporated polygraph testing (*k* = 6), they generated weaker effects than programs that did not contain this element or for whom this element was unknown. While it is possible that use of the polygraph may impede the treatment process and effectiveness, the studies examined did not do direct comparisons of using the polygraph vs. no polygraph within a given program. Given the small *k*, we also do not know if these studies were representative of all programs that employ the polygraph and how other treatment-relevant features of these programs in terms of content and foci compare to others elsewhere.

### Program Staffing

Due to the pressures associated with providing specialist treatments on a large scale and at a low cost, paraprofessionals have been increasingly used to deliver offense-specific psychological interventions as opposed to qualified psychologists, with the latter moving towards a “hands-off” monitoring or supervisory role in some jurisdictions [27, 28]. Gannon and Ward hypothesized that the direct involvement of qualified psychologists in the delivery of treatment should produce more positive effects due to their level of clinical competence and subject-specific expertise [27]. However, little research has examined whether the move towards the use of paraprofessionals has impacted the quality or delivery of treatment. Gannon et al. examined the effects of having direct psychological input in treatment delivery (e.g., “hands-on” facilitation) as well as the presence or absence of supervision for program staff [5••]. They found that offense-focused psychological treatment for sexual offending was most effective when a registered autonomous psychologist was consistently present in facilitating treatment (compared to inconsistently present, unknown, or never present). The presence of supervision was also associated with more positive treatment outcomes; however, the provider of this appeared to be less important, unless psychologists and non-psychologists were both involved in providing this, where treatment outcomes were weaker.

### Treatment Setting

Given that treatment for sexual offending is delivered across a range of contexts (e.g., prisons, therapeutic communities, secure mental health hospitals, and community settings), it is unsurprising that treatment setting has been considered a potential moderating factor. Unlike other moderators that have been discussed so far, research findings relating to treatment setting are more consistent. Overall, psychological interventions delivered in both inpatient and community settings have been found to be associated with reductions in sexual reoffending; however, the majority of meta-analyses suggest that community-based programs appear to produce stronger treatment effects for both sexual and general reoffending [5••, 7, 8 16]. The exception to this is Schmucker and Lösel’s 2015 meta-analysis, which found only community programs significantly reduced sexual reoffending [12].

## What Works Best for Whom?

Until this point, we have focused on features of treatment that have been found to be associated with superior outcomes. However, while large-scale meta-analyses appear to suggest an overarching positive effect for sexual offense treatment that is both sexual offense-specific and psychological, it is important to note that the wider psychotherapy literature suggests that even when group effects for treatment are identified, at an individual level, the same intervention may be more (or less) beneficial for some individuals than others [29]. Given the heterogeneity of individuals with sexual convictions, there are a range of individual factors that may act as potential moderators for treatment including age, offense type, risk level, treatment (non)completion, and level of coercion (i.e., whether treatment is mandated or voluntary). We now consider available research findings for each of these.

Several large-scale meta-analyses have found that treatment for sexual offending produces the strongest effects for clients under 18 years of age [7, 8, 12, 14, 16, 30]. Within adult samples, treatment of age homogenous groups has been found to produce stronger effects [8, 30]. While treatment for sexual offenses appears to significantly reduce sexual reoffending in both men who have offended against children and those who have offended against adults, the strongest effects have been reported for those convicted of adult offenses (e.g., rape and exhibitionism) [8, 30]. Research has not conclusively indicated any significant differences in treatment outcomes for individuals classified as high risk of reoffending compared to those classified as low risk; however, findings do suggest a stronger treatment effect for higher risk individuals [7, 12, 23]. Differences across studies for risk outcome have largely been attributed to the varying ways in which risk categories (e.g., low, medium, and high) have been operationalized [7, 12].

While there has been limited research on whether personal characteristics moderate outcomes for sexual offense treatment, there has been more of a focus on the effects of treatment (non)completion and coercion (e.g., whether treatment is voluntarily attended or mandated). Individuals who commence treatment may not complete for a variety of reasons including motivation; treatment readiness; dissatisfaction with program content, delivery, and format (e.g., working as a group); and the perceived relevance of the intervention [31, 32]. Research from the wider correctional literature suggests that non- or partial completion of treatment is associated with an elevated level of reoffending [33, 34]. This effect has been found to replicate in syntheses of sexual offending treatment, with non-completion of treatment doubling the odds of reoffending [8, 34]. With regard to coercion, findings from the wider literature on correctional treatment programs suggest that mandated treatment in custodial settings is particularly ineffective, whereas voluntary treatment produces significant effects regardless of treatment setting [35]. This effect has been mirrored in meta-analyses exclusively focusing on treatment for sexual offending [8, 12].

## The Influence of Research Design

So far our review has focused on key program and individual factors that have been identified as moderators of treatment success. However, it is also important to consider the role of research design and how this has impacted knowledge proliferation in this area.

### Outcome Measures

Reconviction data (i.e., being convicted by a court of another offense post-treatment) is often used as a long-term outcome measure for treatment success since it aligns with the primary goal of reducing offending behavior and is able to be measured systematically and objectively through routinely recorded police data [36]. However, there has been important discussion in the wider criminological and psychological literature about the accuracy of reconviction as an outcome measure both in the way in which it is recorded and whether it truly reflects actual levels of undetected offending [36, 37]. For this reason, some studies have used broader outcome measures of treatment success (e.g., arrest, reconviction, reoffense, absconding, probation violation, parole violation, parole suspension, parole revocation, and incarceration); however, these also rely on official detection and recording [36]. It is also possible that more liberal operationalizations beyond the threshold of conviction (e.g., rearrest) can represent false positives (e.g., finding of not guilty). Moreover, others have used indexes of offense severity based on the nature and number of new convictions, such as the Cormier-Lang scale, to evaluate treatment effects per a harm reduction model [38]. Few studies have incorporated data from unofficial records or self-reports (e.g., offense-related behaviors). When they do, however, research indicates that these broader outcome measures result in higher reoffending rates [39]. Meta-analyses of treatment for sexual offending have examined the effects of reoffending data quality on treatment outcome and found that studies rated as having high or very high-quality reoffending indicators (e.g., longer follow-up times) are associated with stronger reoffending reductions [5, 12].

### Research Designs

Randomized controlled trials (RCTs) are considered the “gold standard” for intervention evaluation across disciplines. However, it is recognized that it is not always possible to implement RCTs when evaluating treatment for sexual offending due to legal and ethical concerns regarding withholding treatment and/or providing suboptimal treatment to one or more groups within the trial and the potential for community harm [1, 40]. As a result, many evaluations of sexual offending interventions have adopted quasi-experimental designs, comparing outcomes for those who complete treatment to a variety of “untreated” comparison groups (e.g., incidental cohorts, retrospective cohorts, treatment non-completers, treatment decliners, individuals with lower treatment need) [10]. Although quasi-experimental designs represent good quality evaluations (tier below RCTs), international meta-analyses suggest that methodological and contextual characteristics may influence treatment outcomes. For example, sample size, quality of outcome reporting (e.g., source and quality of reoffending data), base rate of reoffending, the definition and quality of the comparison group (e.g., the use of treatment non-completers or decliners), and the use (or non-use) of matching have been found to explain some of the variance in study outcomes [5, 8, 10, 12]. Recently, Lösel et al. examined the effect of different matching approaches (e.g., exact matching and propensity score matching) on treatment outcomes with a sample of 693 men convicted of a sexual offense residing in German prisons [11•]. While results were broadly similar using both methods, different effects were identified for certain types of reoffending. To illustrate, exact matching showed a negative trend for sexual reoffending and propensity score matching a positive effect. Lösel et al. concluded that more high-quality studies, including replications and differentiation studies (e.g., methodological and outcome comparisons), are needed to enable stronger conclusions to be drawn about the wider effect of treatment for sexual offending.

### Staffing

Finally, programs are only as effective as their implementation, and when it comes to implementation, staffing is key. Frontline staff deliver the programs, they are the eyes and ears of an institution, and they are responsible for imparting new prosocial behavioral skills and problem-solving strategies onto their correctional clientele. We see at least two issues here, the first of which is program fidelity. When programs are delivered as they are intended, this is associated with cumulatively larger effects in terms of recidivism reduction [41]. The second issue is staff relationships with clientele. The general responsivity principle can be succinctly stated as follows: “Effective rehabilitative efforts involve workers who are interpersonally warm, tolerant, and flexible, yet sensitive to conventional rules and procedures” [19] (pp. 36-37). There is a large literature demonstrating that the characteristics of staff members and their quality of relationships with correctional clientele impact program outcome. Termed “core correctional practices” (or CCPs), these refer to a constellation of staffing techniques and behaviors that include relationship practices, effective use of authority, prosocial role modeling, effective reinforcement, effective disapproval, and prosocial problem-solving. In a classic meta-analysis, Dowden and Andrews found that correctional programs broadly adhering to RNR had significantly larger effect sizes in terms of recidivism reduction when CCPs were followed (φ = .25, *k* = 75) compared to otherwise “appropriate” programs that did not demonstrate use of CCPs (φ = .16, *k* = 71) [42]. A recent meta-analysis focusing specifically on community supervision officers found officers trained in CCPs had lower rates of recidivism among their probationers than those officers without the training (36.2% vs. 49.9%, respectively, *k* = 10) [43]. In short, a program can be sound in form and content and evaluated with elegant rigorous methodology; however, this all becomes moot without well-trained staff to implement the program with fidelity and adhere to effective and humane relationship practices with their clientele.

## Conclusions

Broadly, recent research suggests that psychological offense-specific treatment for sexual offending has some effect in reducing both sexual and general reoffending and outcomes can be further optimized under certain conditions, for example, adhering to RNR principles, incorporating cognitive behavioral principles, including behavioral reconditioning for inappropriate sexual arousal, having “hands on” involvement from a registered psychologist in the delivery of treatment, providing program staff with supervision, and delivering treatment in community settings. Further, individuals classified as high risk and who engage in treatment voluntarily (i.e., are not mandated to attend) are likely to make the largest gains. In contrast, the use of the polygraph within treatment and having mixed supervision with both a psychologist and non-psychological practitioner have been found to be associated with poorer outcomes. There also appear to be adverse effects of partial or non-completion of treatment consistent with the wider correctional treatment literature. Although the existing research provides us with some indicators of the requirements for treatment success, there is a lack of agreement between studies with regard to certain factors, such as delivery format (e.g., group or individual), timing of treatment, and intensity of treatment, while other treatment factors have been overlooked to date (e.g., homogeneous vs. heterogeneous group membership, therapist qualifications, and therapist to client ratios) [26•]. Future research would benefit from further exploring key ingredients for successful treatment as well as the role of individual characteristics, so as to ascertain “what works best for whom?” In particular, more high-quality differential studies are needed to enable stronger conclusions to be made about the wider effects of treatment, to settle debate around the impact of particular treatment characteristics where current findings are conflicting or inconclusive, as well as to further knowledge development about “what works best for whom?”.

## Footnotes

1 One further meta-analysis has been published since by Harrison et al. [18]; however, this meta-analysis does not include any studies published after 2012.
